# Simultaneous liver T_1_ , T_2_ , and ADC MR fingerprinting using optimized motion‐compensated diffusion preparations: An initial validation on volunteers

**DOI:** 10.1002/mrm.30622

**Published:** 2025-07-09

**Authors:** C. Velasco, C. Castillo‐Passi, N. Chaher, D. C. Karampinos, P. Irarrazaval, A. Phinikaridou, R. M. Botnar, C. Prieto

**Affiliations:** ^1^ School of Biomedical Engineering and Imaging Sciences King's College London London UK; ^2^ Institute for Biological and Medical Engineering Pontificia Universidad Católica de Chile Santiago Chile; ^3^ Millenium Institute for Intelligent Healthcare Engineering Santiago Chile; ^4^ Department of Diagnostic and Interventional Radiology, School of Medicine & Klinikum rechts der Isar Technical University of Munich Munich Germany; ^5^ School of Engineering, Electrical Engineering Department Pontificia Universidad Católica de Chile Santiago Chile

**Keywords:** ADC, diffusion imaging, liver, MR fingerprinting, MRF, multiparametric imaging, T_1_, T_2_

## Abstract

**Purpose:**

To develop a novel MR fingerprinting sequence using optimized motion‐compensated diffusion preparations for simultaneous T_1_, T_2_, and ADC quantification of liver tissue in a single breath‐held scan.

**Methods:**

A radial spoiled gradient echo acquisition with magnetization preparation modules for T_1_, T_2,_ and ADC encoding is proposed. To compensate for the signal voids generated by the diffusion preparation, the combination of (1) a breath‐held scan, (2) peripheral pulse signal triggering, and (3) an optimized motion‐compensated diffusion‐preparation module is employed. Phantom experiments were performed to test the accuracy of the technique. The sequence was evaluated in 11 healthy subjects in comparison to conventional mapping techniques. Additional in vivo repeatability assessment experiments were performed.

**Results:**

T_1_, T_2_, and ADC quantification showed good correlation (r^2^ > 0.9 for all cases) with reference maps in phantoms and good agreement in vivo against clinical scans (bias not significantly different from zero). A peripheral pulse trigger delay of 200 ms was used to reduce cardiovascular motion artifacts. The repeatability tests prove a low interscan coefficient of variation and a high intraclass correlation coefficient of greater than 0.9 for all cases.

**Conclusions:**

Simultaneous quantification of T_1_, T_2_, and ADC in liver tissue in a single MR fingerprinting scan of ˜16 s has been proposed, enabling a comprehensive evaluation of hepatic disease through co‐registered multiparametric imaging. Further studies are warranted to test this approach in patients with suspected diffuse liver disease to evaluate its potential for liver tissue characterization and tumor staging.

## 1 INTRODUCTION

Liver disease accounts for approximately one in every 25 deaths worldwide, with a total of two million deaths annually.^1^, ^2^ Its mortality is mainly associated with hepatocellular carcinoma and cirrhosis, a late stage of diffuse liver disease that is itself considered a major risk factor for hepatocellular carcinoma.^3^ Liver biopsy is the current gold standard for staging and assessing liver fibrosis in diffuse liver disease.^4^ However, biopsies present challenges that can affect the accuracy and diagnostic value of this technique, such as inter‐rater variability due to sampling errors^5^, ^6^ from its intrinsic heterogeneity, patient discomfort due to its invasiveness, and the risk of pain and bleeding.^7^, ^8^ MRI arises as one of the most promising noninvasive techniques to assess liver disease due to its outstanding tissue characterization capability.

Quantitative MRI has shown potential to provide imaging markers for evaluating both diffuse liver disease and focal liver lesions. T_1_ and T_2_ mapping are used for inflammation detection and fibrosis assessment,^9^, ^10^, ^11^ $T_{2}^{*}$ for assessing iron overload,^12^ and proton density fat fraction (PDFF) for hepatic fat content quantification.^13^ These quantitative maps are usually acquired sequentially, which may lead to potential misalignment, bias to inter‐parameter dependencies, and long scan sessions.

Over the past decade, significant efforts have been made to obtain simultaneous quantitative information for hepatic tissue.^14^, ^15^, ^16^, ^17^ Notably, the work of Banerjee and Pavlides et al.^14^, ^15^ led to the development of LiverMultiScan (Perspectum, Oxford, UK), an MRI‐based test that aims to provide a complete picture of liver health and is now being utilized in many clinical studies across multiple cohorts of patients with liver disease.^18^, ^19^, ^20^ Besides this work, other groups have proposed alternative sequences to simultaneously obtain water T_1_, PDFF, and $T_{2}^{*}$. One of these is MR fingerprinting (MRF),^21^, ^22^, ^23^ which can efficiently encode tissue properties using magnetization‐prepared fast undersampled acquisitions. Jaubert et al. showcased T_1_, T_2_, $T_{2}^{*}$, and PDFF liver tissue characterization,^24^ with clinical feasibility investigated in patients with focal liver disease, to differentiate between benign and malignant lesions.^25^ This MRF sequence also showed high repeatability and good agreement with histopathology analysis in a prospective study with 56 participants with diffuse liver disease,^26^ with a short scan time of 15 s.

Quantification of the ADC is also important for liver characterization because it can differentiate between benign and malignant focal liver lesions and determine the stage of liver fibrosis.^27^, ^28^, ^29^, ^30^, ^31^ However, ADC maps have not been included in simultaneous quantitative schemes for liver imaging because the inherent sensitivity of diffusion imaging to motion generates signal voids caused by phase dispersion resulting from the blood flow within the hepatic vessels and the effect of cardiac motion,^32^ which degrades the quantitative maps.

To address these challenges, nonsimultaneous approaches in other organs have used motion compensation techniques such as moment (M)1^33^ and M1‐M2^34^, ^35^ gradient‐moment nulling in addition to respiratory gating. In the case of the liver, its short T_2_ compared to other organs (T_2_ of 40 ms) makes it harder to use the long gradient waveforms required for M1 and M2 moment‐nulling to achieve an adequate diffusion encoding or b‐value. Thus, optimization techniques have been used to reduce the echo time (TE) of such acquisitions.^36^, ^37^

Other approaches do not use motion compensation and rely on long scan times and/or data rejection of corrupted data per heartbeat. Among them, Zhang et al. proposed a stimulated echo–based T_1_, T_2_, ADC mapping sequence^38^ that showed good agreement in phantoms, brain, and prostate scans. Hutter et al. proposed a 3D multi‐echo sequence with efficient positioning or preparation blocks for simultaneous T_1_, $T_{2}^{*}$, and diffusion mapping.^39^ Ma et al. developed a 3D brain T_1_, T_2_, ADC sequence based on MR multitasking^40^ that was evaluated in healthy subjects and three postsurgery patients. There have also been successful attempts in recent years in the MRF field. Recently, Afzali et al. proposed an MRF sequence in which T_1_, T_2_, and ADC maps were derived on phantom and brain scans with scan times of just 24 s per slice.^41^ On the other hand, Cao et al. showed an MRF approach for 1 mm^3^ isotropic 3D T_1_, T_2_, proton density, ADC, and fractional anisotropy mapping in the brain with scan time of 10 min.^42^ Finally, Fan et al. proposed a 6 min MRF sequence for simultaneous T_1_, $T_{2}^{*}$, diffusion, and perfusion brain imaging, which was successfully evaluated in healthy subjects and two patients with subacute ischemic stroke.^43^

However, none of the current approaches are suitable for simultaneous T_1_, T_2_, and ADC mapping of the liver because they do not tackle the inherent signal corruption caused by the significant motion in the liver and the limited scan duration when the acquisition is performed under breath‐hold.

Here, we propose a novel fast radial‐based MRF sequence for simultaneous T_1_, T_2_, and ADC quantification in a single scan of approximately 16 s that can be performed under a breath‐hold. Our proposed approach consists of a series of 16 triggered blocks that enable T_1_, T_2_, and diffusion encoding via magnetization preparation modules applied before each readout. To compensate for the signal voids generated by the diffusion preparation, the combination of (1) a breath‐held scan, (2) peripheral pulse unit (PPU) triggering, and (3) optimized motion‐compensated diffusion‐preparation module is employed. The motion‐compensated diffusion preparation module is designed to achieve optimal diffusion encoding for a set TE, thereby reducing signal‐to‐noise ratio (SNR) loss due to T_2_ decay. The feasibility and repeatability of the proposed approach was evaluated in phantoms and 11 healthy subjects at 3 T.

## 2 METHODS

### 2.1 Sequence details

**FIGURE 1:**
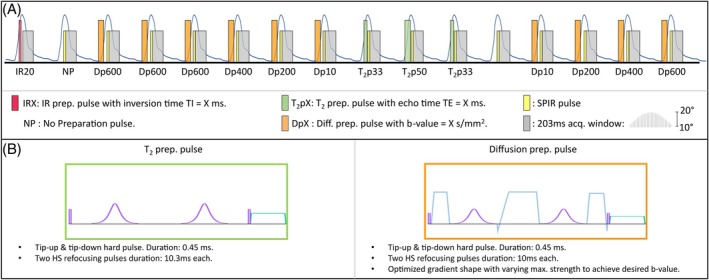
(A) Proposed MRF pulse sequence for simultaneous T_1_, T_2_, and ADC liver quantification. The sequence consists of 16 blocks triggered by PPU signal measured at the subject's finger (blue line). Each acquisition window has a fixed duration of 203 ms, consisting of 30 shots with TR = 7 ms, and is preceded by a preparation pulse to add T_1_ (inversion recovery, red box), T_2_ (T_2_ preparation, green), or ADC (Dp, orange) encoding. T_2_ preparation and Dp pulses are shown in (B). Dp, Diffusion preparation pulse; MRF, MR fingerprinting; PPU, peripheral pulse unit.

A novel gradient echo MRF sequence with tiny golden radial trajectory (spoke rotation angle of 23.6281°) and varying magnetization preparation pulses is proposed and is schematically shown in Figure 1A. The acquisition is triggered with PPU to further decrease cardiovascular motion‐related signal corruption. The sequence consists of 16 blocks of 30 RF shots, each with varying flip angles in the range 10°–20° and constant TEs and TRs. One acquisition block is preceded by an inversion recovery pulse with inverstion time = 20 ms; three blocks are preceded by T_2_ preparation pulse (T_2_p(repetitions) = {33(2), 50(1)}ms, containing two hyperbolic secant refocusing pulses) for T_2_ encoding; 10 blocks are preceded by isotropic diffusion preparation pulses (b‐values(repetitions) = {10(2), 200(2), 400(2) and 600(4)} s/mm^2^); and two blocks are left without preparation to allow for magnetization recovery.

### 2.2 Diffusion‐preparation pulse optimization

**FIGURE 2:**
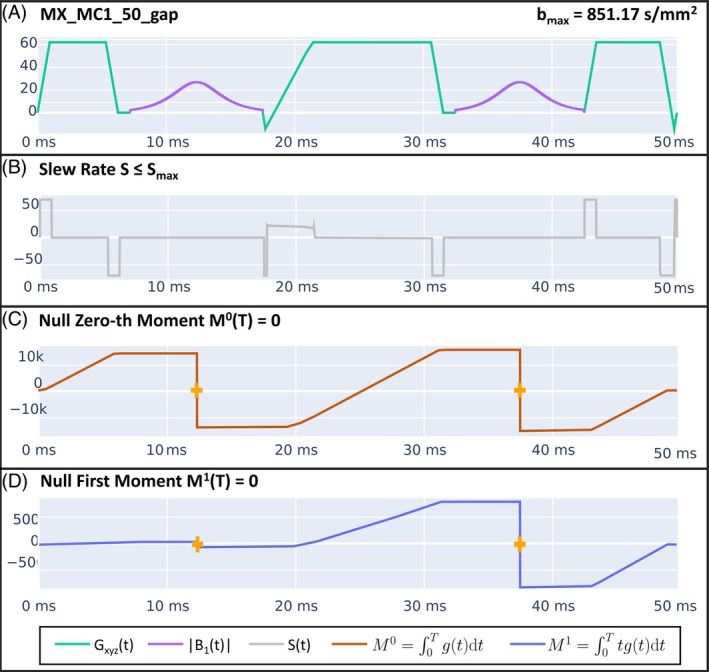
(A) Dp module employed in the proposed T_1_, T_2_, and ADC liver MRF. Optimization was performed considering certain restrictions, including slew rate below a fixed threshold to match PNS limitations, and nulling of M0 and M1 moment to make the preparation more robust against cardiac motion and vascular flow during the preparation time. (B), (C), and (D) show the slew rate, zero‐th moment, and first moment of Dp depicted in (A). The slew rate restriction was applied during the whole preparation, whereas M0 and M1 were set to be null at t = T, the time at which the preparation ended. M0, zeroth moment; M1, first moment; PNS, peripheral nerve stimulation.

The diffusion preparation pulse (Dp) was based on a T_2_‐preparation pulse (Figure 1B), including a set of gradients between the RFs to add diffusion encoding (Figure 1C). The duration of the Dp was fixed to 50 ms, which provided a reasonable T_2_ decay and maximum b‐value for liver tissue, including two adiabatic refocusing pulses of 10.3 ms to increase robustness against high field inhomogeneities. The pulse was designed to reach the highest possible b‐value for a given diffusion preparation duration based on the hardware's maximum gradient and slew‐rate specifications. Gradients were applied simultaneously along the three Cartesian axes to increase the achievable maximum b‐value, and were scaled when smaller b‐values were desired. The gradient waveforms were optimized to achieve M0 and M1 gradient moment nulling for increased robustness against cardiac motion and vascular flow (Figure 2), also compensating for concomitant gradients effect. Optimization was performed using the Julia^44^ JuMP.jl^45^ package for mathematical optimization with an interior point solver Ipopt.jl.^46^ Short eddy currents were alleviated by leaving a 1 ms gap between each gradient lobe and its consecutive RF so they would not generate tagging effects when combined with the RF.

**FIGURE 3:**
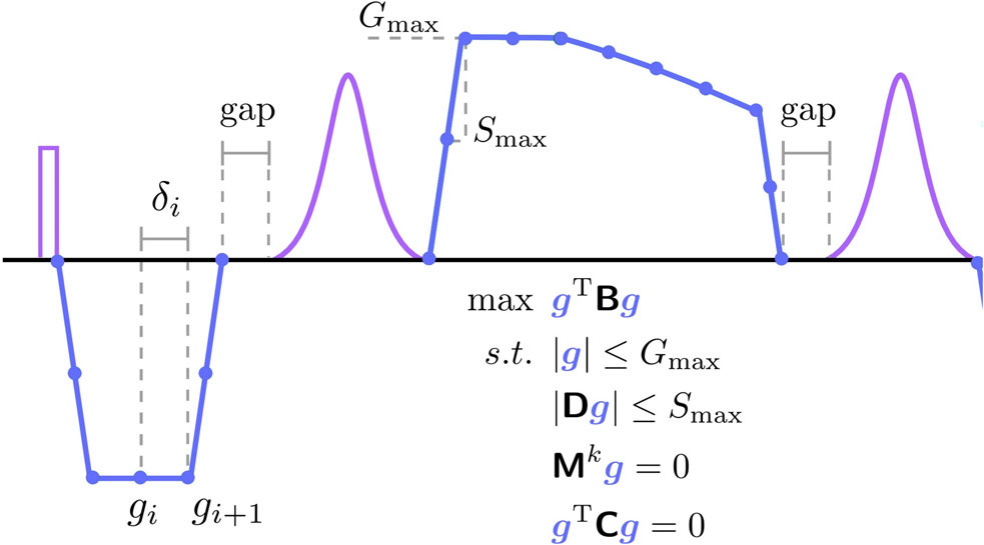
Diffusion gradient optimization. Gradients were optimized in the diffusion preparation module by assuming piecewise‐linear functions. The gradient nodes are represented by blue points and the resulting gradients by a blue line. The RF waveforms for the excitation and the hyperbolic secant adiabatic refocusing is in purple. A quadratic optimization function for the calculation of the b‐value was used. As constraints, maximum gradient, maximum slew rate, M0 and M1 moment nulling, and concomitant gradients nulling were included. Starting point of the gradient nodes g_i_ was set to G_max_.

The optimization problem was formulated efficiently by modeling the gradient waveforms as piece‐wise linear functions. The continuous problem defined by the gradient nodes can be solved by assembling matrices (code available in https://github.com/cncastillo/DiffPrepMCMRF) that can later be used directly in a conventional optimization solver such as Ipopt (Figure 3). Reducing the problem to a small number of gradient nodes (g_i_, separated by four dwell times = 25.6 μs) in order to then linearly interpolate the results to the hardware's dwell time of 6.4 μs increased the optimization speed without losing any of the moments or the concomitant gradients guarantees. The starting point of the gradient nodes g_i_ was set to G_max_.

### 2.3 MRF reconstruction and T_1_ , T_2_ , ADC map estimation

A low‐rank inversion approach^47^ with dictionary‐based singular value decomposition,^48^ regularized with a multi‐contrast high dimensionality undersampled patch‐based reconstruction algorithm (HD‐PROST),^49^ was used to reconstruct the highly undersampled acquired images (HD‐PROST self‐similar patches = 25; patch window = 25; patch size = 5 × 5; regularization term λ = 3 × 10^−3^). The rank of the compression was R = 7, determined by the minimum value that captured 96% of the matrix energy ratio of the singular value decomposition. The reconstruction problem was solved via the alternating direction method of multipliers as previously described^50^ (alternating direction method of multipliers iterations = 3; conjugate gradient iterations = 4). Coil sensitivity maps were derived from ESPIRiT^51^ using all the acquired data, and density compensation functions of the radial readout sampling were calculated via Voronoi diagrams.

T_1_, T_2_, and ADC maps were obtained by matching the reconstructed images against a dictionary of simulated signal evolutions. Extended phase graphs^52^ were employed to simulate each dictionary entry. The diffusion effects introduced by any gradients used in the sequence (readout spoilers, magnetization preparation spoilers, and diffusion preparation) were included in the formalism.^53^ No slice profile, B_0_ and B_1_ inhomogeneities, or magnetization transfer effects were considered in this calculation.

Reconstruction was performed with an in‐house developed software (MatLab R2021a; MathWorks, Natick, MA) on a Linux workstation (Intel Xeon E5‐2697 at 2.3 GHz, 36 dual‐thread cores), and took ˜15 min. The subject‐specific extended phase graphs dictionary generation took 5–15 min depending on the number of entries calculated (15k to 100k).

### 2.4 Experiments

All scans were performed on a clinical 3 T scanner (Achieva TX 3.0 T, Philips, Best, The Netherlands) using two 16‐channel Sense XL torso coil. Written informed consent was obtained from all subjects prior to imaging, and the study was approved by the institutional ethics committee. In vivo acquisitions were performed on the end‐expiration phase of a breath‐hold and triggered using a PPU placed on the subject's index finger. The PPU trigger delay was optimized on two subjects by inspecting the zero‐filled images of each heartbeat for MRF scans performed with trigger delays between 200 and 450 ms. The trigger delay showing the least amount of artifacts was considered for the remaining experiments described.

Acquisition parameters for both phantom and in vivo studies include: FOV = 320 × 320 mm^2^ at 1 × 1 mm^2^ resolution, 6 mm slice thickness, TE = 3.3 ms, TR = 7.0 ms, bandwidth = 275 Hz/px, and acquisition time ˜ 16 s. A reconstruction voxel size of 2 × 2 mm^2^ in‐plane was used.

The hardware and software limitations of the gradient system of the employed scanner (Quasar Dual gradients) were max. amplitude for each gradient axis G_max_ = 62 mT/m with max. slew rate for each axis S_max_ = 100 T/m/s. S_max_ was further restricted to 70 T/m/s to satisfy peripheral nerve stimulation (PNS) safety thresholds.

### 2.5 Phantom experiments

The proposed approach was evaluated in two different phantoms: (a) a standardized T1MES^54^ for T_1_, T_2_ validation; and (b) a set of tubes filled with aqueous solutions of varying concentration of polyvinylpyrrolidone (10%–50% w/v polyvinylpyrrolidone K30, CAS‐No. 81420 Sigma Aldrich, St. Louis MO) to generate a range of isotropic diffusion values.^55^ A fixed concentration of NiCl_2_ (2 mM, Sigma Aldrich, St. Louis, MO) and Agarose (2% v/v, Sigma Aldrich, St. Louis, MO) was also added to these dilutions to obtain physiological T_1_ and T_2_ values (T_1_ ≈ 700 ms and T_2_ ≈ 40 ms). Measurements from the proposed T_1_, T_2_, and ADC liver MRF scans were compared against conventional spin‐echo (SE) references: T_1_ inversion recovery SE for T_1_ mapping, multi‐echo SE for T_2_ mapping, and DWI multishot (mSh) SE for ADC mapping. Conventional scans that are usually performed in vivo were also performed for comparison: T_1_ modified Look–Locker imaging (T_1_‐MOLLI),^56^ T_2_ gradient and SE (T_2_‐GRaSE),^57^ and single‐shot DWI‐SE for T_1_, T_2_, and ADC respectively. Further details on these sequences are shown in Table S1.

Two dictionaries were used for the phantom scans, one for the T1MES and a second one for the diffusion phantom. The dictionary generated for T1MES phantom studies covered the following parameters of interest: range of T_1_ values were [50:50:900, 900:25:1400, 1400:200:3000] ms; range of T_2_ values were [5:5:30, 30:2:80, 80:20:200, 200:100:600] ms; and range of ADC values were [0.0:0.05:2.5, 2.5:0.5:5.0] × 10^−3^ mm^2^/s. Because the diffusion phantom was designed to provide values similar to those expected in liver tissue, the dictionary employed contained the following parameters of interest: T_1_ values [100:100:500, 500:40:1000, 1000:200:3000] ms, T_2_ values [6:3:60, 60:20:100, 100:50:300] ms, and ADC values [0.1:0.1:2.5, 3.0:1.0:5.0] × 10^−3^ mm^2^/s.

### 2.6 In vivo experiments

The sequence was evaluated in eleven healthy volunteers (five female, mean ± SD age = 30.1 ± 3.7 years). The proposed T_1_, T_2_, and ADC liver MRF scans were performed on axial view, and the MRF‐derived parametric maps were compared against conventional individual single breath‐hold maps (T_1_‐MOLLI and T_2_‐GRaSE, 2 × 2 × 6mm) and free‐breathing DWI‐SE (3 × 3 × 4mm). The DWI‐SE sequence consisted of a single‐shot respiratory gated scan, with {b‐values(averages)} = {0(1), 50(1), 300(2), 600(3), 800(4)} s/mm^2^. Additional details are shown in Table S1. Furthermore, three separate scans were acquired within the same scanning session on 10 out of 11 volunteers to assess scan–rescan repeatability. All subjects were asked to remain in the same position between scans. The subject‐specific dictionary generated for in vivo studies was the same used in the diffusion phantom studies.

### 2.7 Image analysis

In phantom studies, quantification was made for each sample within a 15 mm‐diameter circular region of interest (ROI). MRF T_1_, T_2_, and ADC measurements within each ROI, determined as mean ± SD, were compared against their respective reference by Pearson correlation, where coefficients of determination and best fits are reported.

For in vivo evaluation of the proposed MRF quantification, T_1_, T_2_, and ADC values were measured for each subject in eight 10 mm‐diameter ROIs manually placed in the liver. Special care was taken to avoid contamination from visible large vessels and organ edges, and covering the whole liver, following Couinaud classification (two ROIs in segments VI/VII, two in V/VIII, two in IVa/IVb, and the last two in segments II/III). The same ROIs were used for MRF‐T_1_, MRF‐T_2_, and MRF‐ADC. Eight ROIs of same size and similar position were placed individually on the T_1_‐MOLLI, T_2_‐GRAaSE, and DWI‐SE reference maps. Agreement between MRF and the reference scans was assessed with Bland–Altman plots,^58^ where limits of agreement were set and reported as the 95% confidence interval (mean ± 1.96 SD).

Intra‐session test–retest repeatability was assessed using the repeated MRF scans of 10 healthy subjects. Mean coefficients of variation (CV) for T_1_, T_2_, and ADC, defined as the average of the ratio of the SD of the measurement within each segment over their mean, are reported. Furthermore, intraclass correlation coefficients^59^ were calculated for T_1_, T_2_, and ADC test–retest intrarater evaluation, assuming a two‐way mixed effect, absolute agreement, and multiple raters/measurements.^60^

Significance level of was set at 0.05. All statistical analyses were performed on GraphPad Prism v8.2.1 (GraphPad Software, San Diego, CA). Data generated and analyzed during the study are available from the corresponding author upon reasonable request.

## 3 RESULTS

### 3.1 Effect of gradient compensations in image artifacts

**FIGURE 4:**
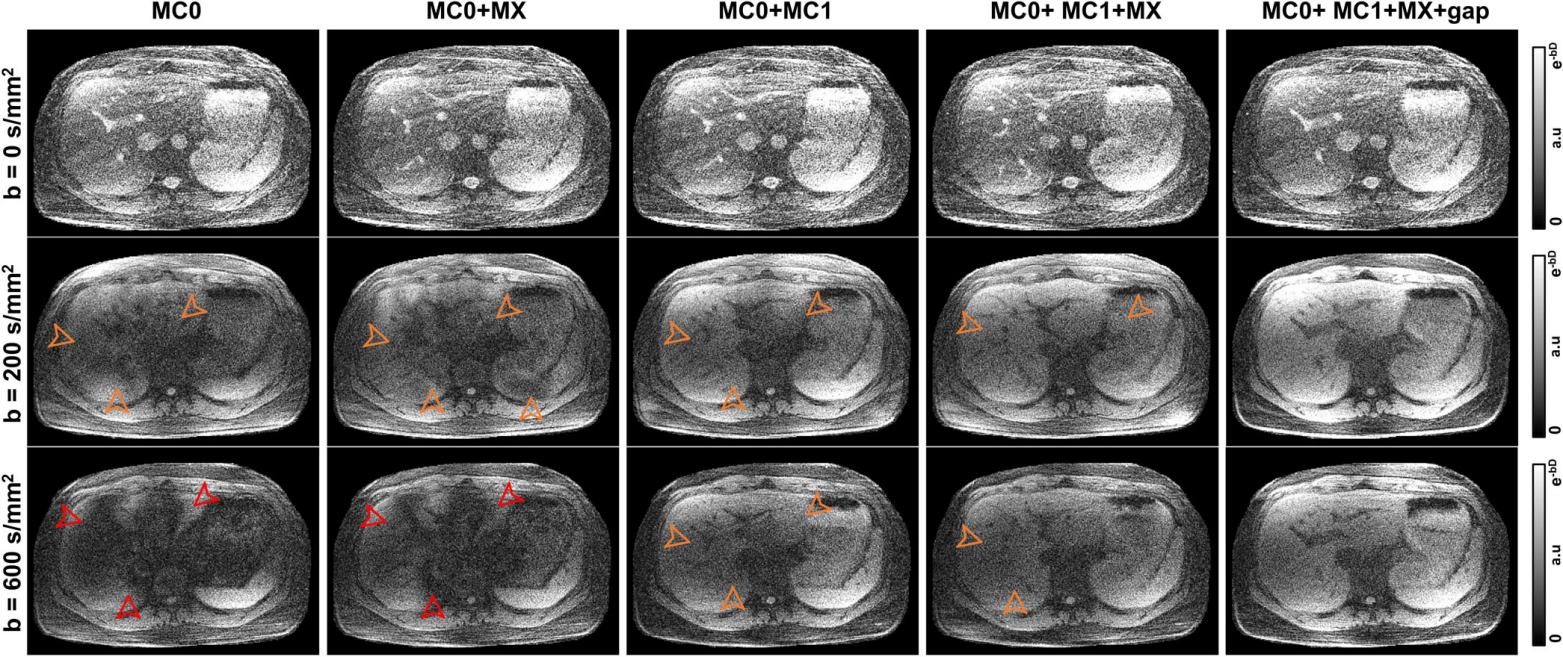
Representative examples of zero‐filled images of one heartbeat after applying a Dp module of b = 0 s/mm^2^ (i.e., no preparation, top row), b = 200 s/mm^2^ (middle row), and b = 600 s/mm^2^ (bottom row). The number of optimization constraints of the Dp module is increasing from left to right. MC0: zeroth moment nulling, MC0 + MX: MC0 with concomitant gradients compensation, MC0 + MC1: zeroth and first moment nulling, MC0 + MC1 + MX: zeroth and first moment nulling with concomitant gradient compensation, MC0 + MC1 + MX + gap: MC0 + MC1 + MX with extra delay of 1 ms after gradients for eddy current compensation. Orange and red arrowheads denote some mild and strong signal dropouts observed. All images are normalized between 0 and e^−bD^ to remove visual effects of signal loss due to diffusion effects and better depict signal dropouts independently of the b‐value of the preparation gradient. Schematic drawings of the Dp modules are shown in Figure S1

Representative examples of zero‐filled images of one heartbeat after applying a diffusion preparation module for different constraint combinations of the Dp gradient waveform are shown in Figure 4. The optimization constraint combinations include MC0: zeroth‐order moment nulling, MC0 + MX: zeroth‐order moment nulling with concomitant gradients compensation, MC1: zeroth‐ and first‐order moment nulling, MC1 + MX: zeroth‐ and first‐order moment nulling with concomitant gradients compensation, and MC1 + MX + gap: MC1 + MX including an additional delay of 1 ms after gradients for eddy current compensation (Figures 2 and 3). Schematic drawings of all the Dp modules are shown in the Figure S1.

Significant artifacts are observed in the images in which diffusion preparation modules had a lower level of gradient compensations. These artifacts are stronger at higher b‐values and disappear on the most compensated pulse, that is, MC1 + MX + gap (right column in Figure 4). This optimized waveform was the one utilized during the rest of the experiments.

**FIGURE 5:**
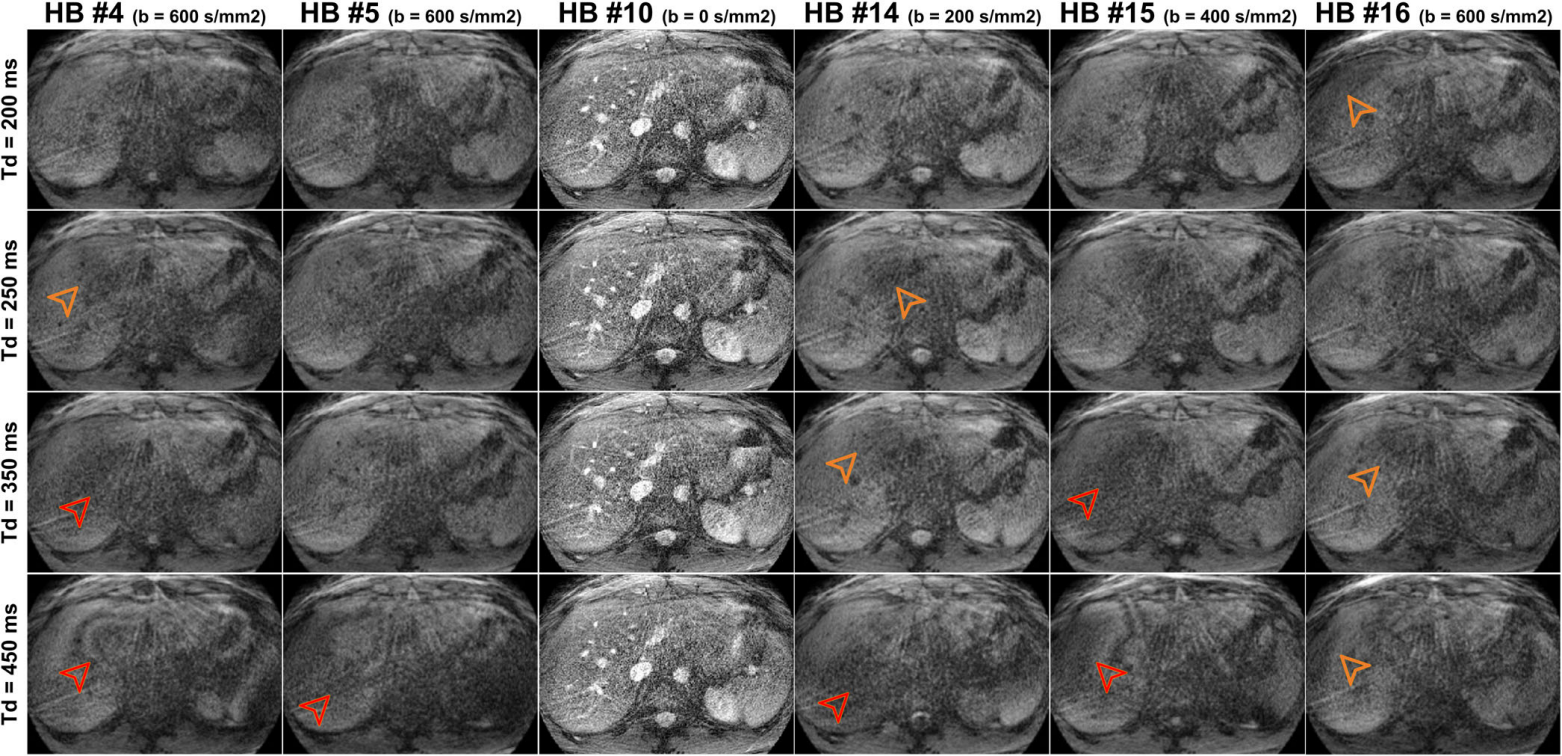
Zero‐filled images of different heartbeats of the proposed MRF scan at different PPU trigger delays for the same representative subject. Heartbeats 4, 5, 14, 15, and 16, corresponding to columns 1, 2, 4, 5, and 6 of the panel, correspond to heartbeats acquired right after a Dp pulse of the b‐value detailed on the header of each column. Heartbeat 9 (column 3) corresponds to the image acquired right after a T_2_p pulse of 50 ms, the same duration as the Dp pulses, which is essentially a Dp pulse of b‐value = 0 s/mm2. Orange and red arrowheads denote some mild and strong signal dropouts observed

Furthermore, as detailed in the Methods section, all MRF scans were performed under PPU triggering, with trigger delay = 200 ms, chosen from the trigger delay optimization experiments. Figure 5 shows a representative case of the same sequence performed at different trigger times on a subject with a heart rate of 62 ± 5 beats per min. Zero‐filled images of different heartbeats of the proposed MRF sequence are shown to demonstrate the trigger effect independently of the regularized reconstruction.

### 3.2 Phantom study

**FIGURE 6:**
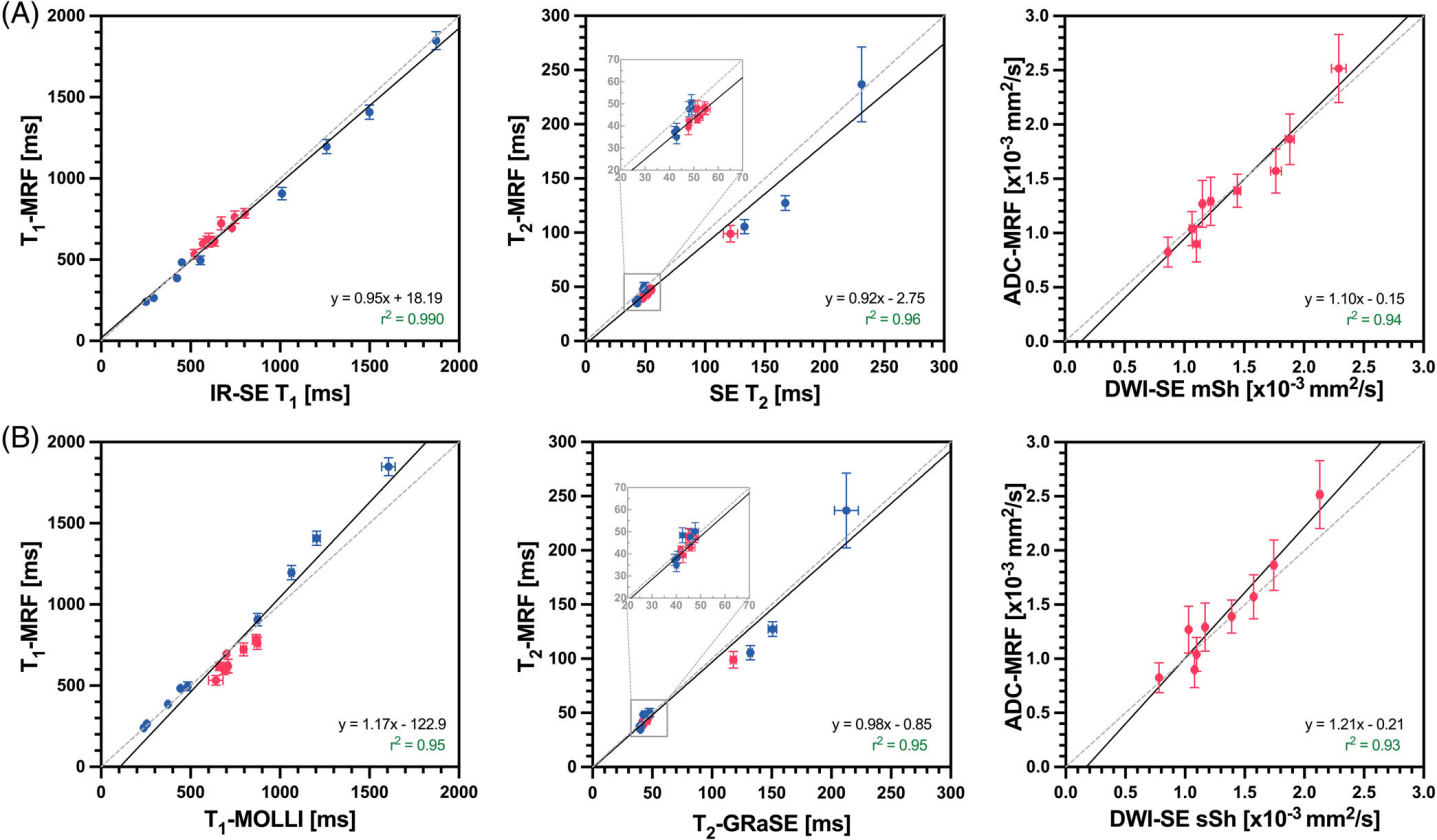
Scatter plots of phantom T_1_, T_2_, and ADC MRF quantification compared with their reference values obtained from long SE reference scans (A: IR‐SE T_1_, multiecho SE T_2_, and DWI‐SE mSh), and shorter clinical maps that were further employed as in vivo reference scans (T_1_‐MOLLI, T_2_‐GRaSE, and DWI‐SE sSh). Quantification was performed inside circular ROIs of 15 mm diameter for every sample two of the two phantoms scanned (T1MES phantoms: blue dots, diffusion phantom: red dots). GRaSE, gradient and spin echo; IR, inversion recovery; mSh, multi shot; ROI, region of interest; SE, spin echo; sSh, single shot.

The quantitative comparison between T_1_, T_2_, and ADC values obtained from the proposed liver MRF approach against reference sequences are shown in Figure 6. Two comparisons are made: first against SE sequences, where maximum accuracy of the reference quantification is sought; and second, against clinical sequences for abdominal parametric mapping.

Evaluation against SE (Figure 6A) shows excellent correlation in all cases (r^2^ = 0.99, r^2^ = 0.96 and r^2^ = 0.94 for T_1_, T_2_, and ADC quantification). Additionally, F tests were performed to determine whether slope could be considered significantly different from one. Correlation of MRF‐ T_1_, T_2_, and ADC is also very good against the clinical reference scans (Figure 6B), with r^2^ = 0.95 for T_1_‐MOLLI, r^2^ = 0.95 for T_2_‐GRaSE, and r^2^ = 0.93 for DWI‐SE. In this case, only in the T_1_‐MRF versus T_1_‐MOLLI slope resulted significantly different from 1 (*p* = 0.02). Bland–Altman analysis for agreement between MRF and their references are shown in Figure S2. Additionally, the parametric maps obtained from MRF and the clinical reference scans, for which quantification is shown in Figure 6B, are also provided in Figure S3.

### 3.3 In vivo evaluation

**FIGURE 7:**
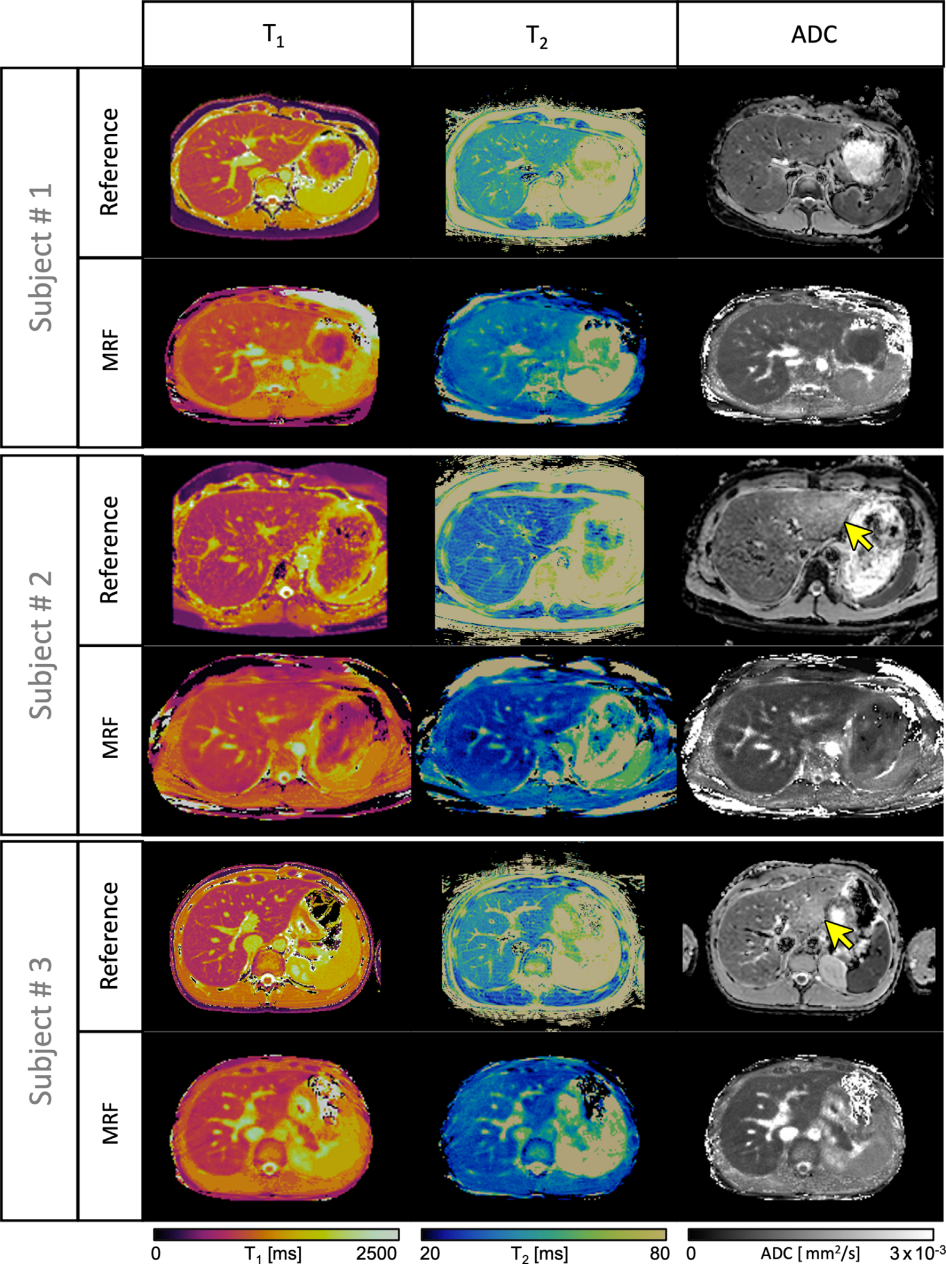
Abdominal axial views of T_1_, T_2_, and ADC quantitative maps in three representative healthy subjects. Reference maps (top row for each subject) of T_1_‐MOLLI, T_2_‐GRaSE, and SE‐DWI are compared against the proposed simultaneous T_1_, T_2_, and ADC MRF maps (bottom row for each subject). Yellow arrows point to abnormally elevated ADC values on reference maps (known artifacts due to cardiac motion), that are not observed in MRF

**FIGURE 8:**
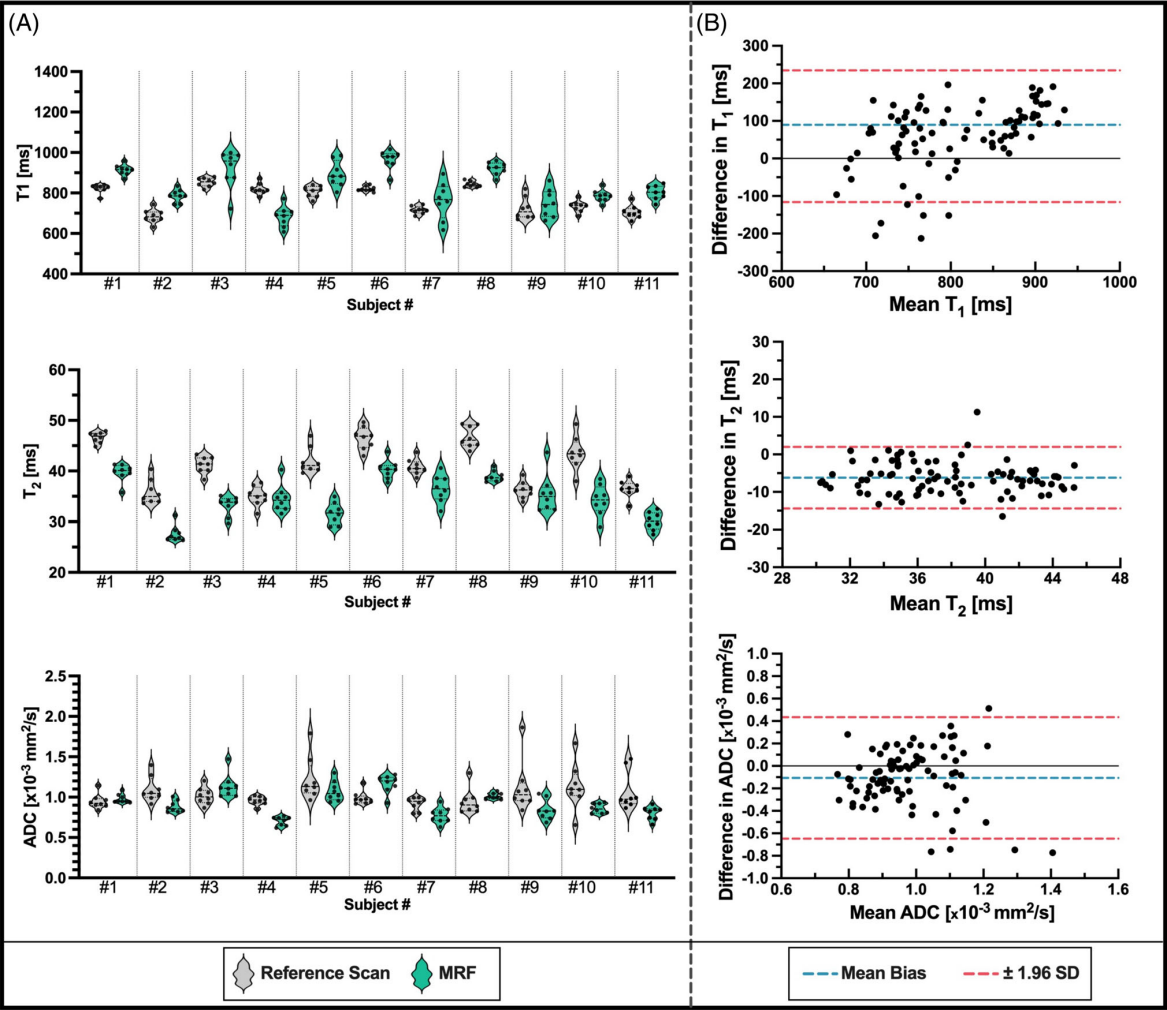
(A) Violin plots of T_1_, T_2_, and ADC MRF quantification (green) in vivo against their reference values (gray) obtained from T_1_‐MOLLI, T_2_‐GRaSE, and SE‐DWI respectively. (B) Bland–Altman plots corresponding to T_1_ (top), T_2_ (middle), and ADC (bottom) quantification within same ROIs shown in (A). Quantification on each volunteer was performed on eight 10 mm‐diameter placed over eight different locations of the liver, placed manually to avoid contamination from blood within visible vessels.

Examples of the co‐registered T_1_, T_2_, and ADC maps obtained from the proposed liver MRF sequence are shown in Figure 7 for three representative healthy subjects. MRF maps are compared to their respective T_1_‐MOLLI, T_2_‐GraSE, and DWI‐SE reference scans, performed as three sequential scans in different breath‐holds. A quantitative evaluation of the agreement between MRF‐derived and reference quantitative maps was performed on the entire study cohort. Figure 8A shows violin plots of quantification within eight different ROIs placed in the liver. A slight overestimation of T_1_‐MRF against T_1_‐MOLLI values is observed, as well as underestimation of T_2_‐MRF against T_2_‐GRaSE. As for ADC quantification, no significant bias was found, but higher variation of ADC values can be found on the reference DWI‐SE scan on some individuals, corroborating that the Dp optimization was able to reduce ADC variability due to reducing motion‐related artifacts. Figure 8B shows Bland–Altman analyses of all the measured values. A positive bias (mean ± SD) of 59.1 ± 89.5 ms for T_1_ measurements was found, with limits of agreement (LoA, 95% confidence interval) = [−116.3, 234.4] ms. For T_2_ measurements, a bias of −6.22 ± 4.19 ms (LoA = [−14.4, 2.0]) was measured. For ADC evaluation, bias was −0.11 ± 0.28, LoA = [−0.65, 0.43]. For all the three agreement analyses performed for T_1_, T_2_, and ADC quantification, a correlation analysis was performed between the mean of quantifications and their difference to analyze a possible significant trend in bias. The slope of these correlations was not significantly different from the null hypothesis of slope = 0 in any Bland–Altman distribution, meaning the biases found neither increased nor decreased with higher (or lower) values.

### 3.4 In vivo repeatability

**FIGURE 9:**
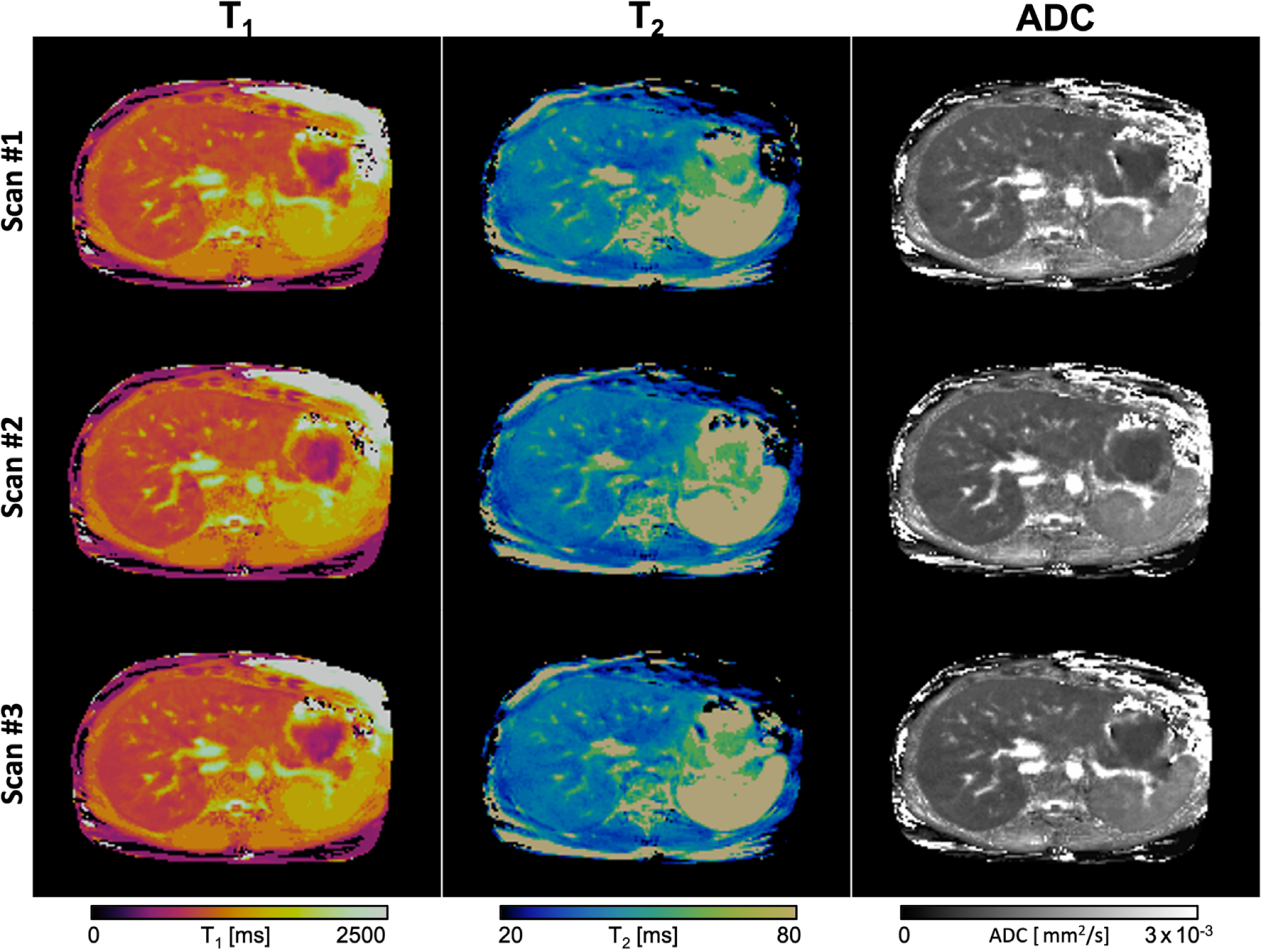
Test–retest repeatability study. Abdominal axial views of the proposed T_1_, T_2_, and ADC MRF quantitative maps in three separate scans from the same representative healthy subject.

**FIGURE 10:**
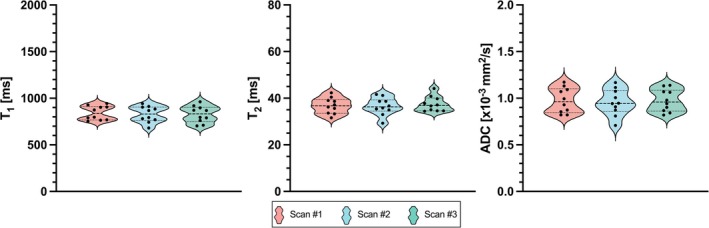
Violin plots of distributions of the proposed T_1_, T_2_, and ADC MRF quantification on every scan of the test–retest repeatability study on ten healthy subjects. Each dot denotes the mean value of the parameter of interest over the eight ROIs drawn within the liver of each healthy subject.

A representative example of the in vivo intrasession repeatability analysis is shown in Figure 9, where the T_1_, T_2_, and ADC maps obtained from three different MRF scans are included. Figure 10 shows the distributions of T_1_, T_2_, and ADC for each one of the three measurements performed in the repeatability study. As shown on Figures 9 and 10, the image quality and quantification on each scan is almost identical. For each parametric map of every subject of the cohort, the quantitative values inside the eight ROIs placed within the liver were calculated, with no significant intrasession differences between groups observed in any case (Figure 10). Additionally, violin plots of each quantification are shown in Figure S4, and CVs among measures are detailed in Table S2.

## 4 DISCUSSION

In this work, we proposed a novel MRF sequence able to produce simultaneous T_1_, T_2_, and ADC maps of the liver with high in‐plane acquisition resolution of 1 mm^2^ in a single ˜16 s breath‐hold scan. Results demonstrate the feasibility of the proposed approach in phantoms and healthy subjects, showing excellent agreement between the MRF maps and their respective reference scans both in phantoms and in vivo. The intrasession repeatability of the quantitative results was also demonstrated by running the same proposed sequence at three independent times during the in vivo scanning session, resulting in a CV of the measured of less than 4% for all three parameters mapped (T_1_, T_2_, and ADC).

The proposed sequence is short enough to be performed under a single breath‐hold (acquired over 16 heartbeats) on the end‐expiration phase, which allows alleviation of the strong motion artifacts produced by the movement on the abdomen due to breathing. This is true for any scan in which abdominal motion is involved but is especially important in the case of liver diffusion weighted imaging (DWI).^61^, ^62^, ^63^ Another source of error in diffusion imaging is cardiac motion, particularly in the left lateral hepatic regions (segments II and III) that are closer to or in contact with the heart.^64^ If this motion happens during the diffusion preparation, the phase dispersion introduced by the gradients leads to signal loss and thus overestimation of ADC values.^65^, ^66^ This overestimation can potentially be reduced by prospective cardiac triggering, as has been shown for liver^66^ and brain^41^, ^42^, ^67^, ^68^, ^69^ imaging. Consequently, PPU triggering was employed in this study to minimize errors induced by cardiac motion.

The optimized Dp had gradient waveforms that achieved the maximum b‐value for a given preparation length of 50 ms, which produces a reasonable T_2_ decay for liver tissue. This contrasts with previously optimized SE‐based approaches,^36^, ^37^ for which preparation requires a dead‐time in which no gradients are applied, thus increasing the TE. Moreover, these waveforms were flow‐ and Maxwell‐compensated while respecting the hardware limits. To alleviate the effect of short eddy currents, we introduced a 1 ms gap between the gradient lobes and the refocusing pulses. Otherwise, eddy current can generate tagging‐like banding artifacts in the presence of cardiovascular flow (Figure 4). For further flow compensation, the acquisitions were synchronized with the PPU signal from a PPU unit, and the trigger delay was adjusted experimentally (Figure 5). The pulse robustness to flow‐ and motion‐related artifacts was successfully shown in vivo (Figure 4), and the accuracy of the MRF‐derived ADC maps was shown in a phantom (Figure 6). Flow compensation of the gradient waveforms was sufficient to eliminate most artifacts.

In vivo quantitative maps (Figure 7) showed good agreement and comparable image quality between the MRF‐derived maps and their respective reference scans. Regarding ADC quantification, there are two major differences between MRF and reference scans. Firstly, yellow arrows in Figure 7 point to areas within the left lobe of the liver that show incorrect overestimation of ADC in the reference scans due to cardiac motion. This artifact is, however, not observed in the proposed ADC‐MRF maps due to optimized diffusion preparation module employed, which has been shown to be more robust against the cardiac motion present in this area. Secondly, quantification within blood vessels present in the liver is noticeably different between MRF‐ADC and DWI‐SE. Though SE sequences provide a black‐blood contrast (see reference ADC maps in Figure 7), this is not the case in our gradient echo MRF sequence because the signal within vessels is not nulled in the heartbeats, whereas there is no diffusion‐preparation module (see Figure 4). Moreover, the Dp waveforms are flow‐compensated, which avoids signal loss due to cardiovascular motion during the preparation, thus obtaining ADC values within the vessels larger than those seen on their respective DWI‐SE reference maps.

The reliability and motion‐robustness of the proposed diffusion preparation was fundamental for the MRF dictionary matching to work properly because one corrupted heartbeat heavily biased the mapping results. Other methods rely in data redundancy by using long acquisitions, data rejection, or signal averaging. These approaches were not easily replicated in the liver using an MRF technique because the liver is affected by respiratory (and cardiac) motion. Unlike with other contrasts, the data corruption caused by motion during the diffusion encoding is difficult to correct as a post‐processing step, or by inclusion of motion compensation techniques in the reconstruction. Thus, we sought to minimize all sources of motion by doing (1) a breath‐held acquisition, (2) with PPU triggering, and (3) motion‐compensated gradients in the diffusion preparation.

The maximum b‐value achieved after gradient optimization by the Dp module was 851 s/mm^2^ for the waveform with M0‐M1 moment and Maxwell compensation. The gradient strength was scaled to obtain b = 200–600 s/mm^2^, achieving different diffusion weightings for the Dp preparations. Phantom and in vivo quantification results show that the range of b‐values was enough to quantify ADC accurately with the proposed approach, despite higher b‐values currently used in the clinical setting in DWI scans. Nevertheless, the use of higher b‐values can be investigated in future work. To enable even higher b‐values, the use of M1‐optimized diffusion imaging^36^ could be explored. This technique considers gradients to be optimized at small non‐zero M1 moments enabling higher b‐values, or conversely, the reduction of the gradients strength, potentially reducing eddy current, slew rate, or peripheral nerve stimulation problems.

To the best of our knowledge, this is the first work to propose an MRF sequence that jointly maps essential relaxation parameters (T_1_ and T_2_) and diffusion (ADC) in the liver within a single scan, incorporating a motion‐compensated diffusion preparation module. This MRF sequence may be extended to incorporate additional parameters of interest. A feature of the proposed sequence is the use of radial readouts, which are inherently robust to motion and could be extended to multi‐echo acquisitions, thus enabling the characterization of two additional parameters: PDFF and $T_{2}^{*}$.^24^, ^70^ Abdominal T_1_, T_2_, $T_{2}^{*}$, FF MRF has shown potential in the characterization of diffuse and focal liver disease, validated in patients and against histological samples as ground truth.^25^, ^26^ The addition of diffusion information to such sequence should enhance the potential of multiparametric imaging to provide a fully comprehensive noninvasive characterization of liver tissue. Moreover, the high resolution achieved with the proposed sequence, and the robustness against cardiovascular motion artifacts due to the optimized diffusion preparation module, could potentially enable extension to other abdominal organs, such as the prostate. T_1_, T_2_ prostate MRF is an active field of research.^71^, ^72^, ^73^ ADC information has been shown to be of great importance for characterization of prostate disease by Yu et al., where they proposed a mixed approach of T_1_, T_2_ MRF plus a separate diffusion scan for prostatic tissue characterization.^74^

This study presents several limitations. The proposed sequence enables 2D mapping, with one slice being acquired per breath‐hold. However, extension to free‐breathing 3D MRF would be beneficial to map the whole liver. 3D T_1_, T_2_, ADC MRF approaches have been previously proposed for brain mapping. The level of complexity of a 3D MRF sequence is higher in the abdominal and thoracic regions, where the bulk motion is added to the respiratory and cardiac motions. Nevertheless, many free‐running 3D MRF approaches have been proposed for 3D whole heart and liver imaging,^23^, ^75^, ^76^, ^77^, ^78^, ^79^ and future work is warranted to use that previous knowledge to convert this 2D approach into a whole‐liver 3D sequence. Moving from 2D to 3D, and from breath‐held to free breathing acquisitions would also allow to increase SNR and quantitative map quality and decrease quantitative map blurring at the expense of a slightly higher acquisition time. This would reduce the streaking artifacts that can be observed in some maps, (e.g., subject 3 in Figure 7), due to the extremely high acceleration factors that must be applied in order to fit acquisition time within a breath‐hold. The use of alternative regularization methods, such as total variation, could mitigate streaking artifacts that are preserved by the locally low‐rank regularization used in this study.

Furthermore, the current implementation of our method is affected by insufficient breath‐hold conditions, which are common in clinical scans because through‐plane motion can alter signal evolution and thereby degrade mapping quality.

In this study, the imaging parameters (e.g., flip angles, TRs, positioning of the preparation modules) were chosen empirically and after a heuristic search over a limited number of combinations. Sequence optimization with Cramér–Rao lower bound^80^, ^81^ could be investigated in the future to further improve the encoding and efficiency of the proposed sequence. Furthermore, reconstruction was performed offline and took about 15 min; thus, future work should investigate speeding up the reconstruction to improve clinical feasibility. With respect to the phantom assessment, we acknowledge that the designed phantom experiments do not replicate motion or flow; their purpose is to validate signal behavior under controlled conditions and quantify diffusion preparation effects in a reproducible environment that presents a wide representation of physiological T_1_, T_2_, and ADC. However, the claim that the proposed sequence is more robust than traditional diffusion scans in the liver could not be confirmed with the utilized phantom.

The proposed approach is applicable to tissues with isotropic diffusion such as the liver because, despite changing the diffusion weighting by adjusting the b‐value, the gradient direction was not modified (all Dp point to [1, 1, 1]). Varying the gradient direction between diffusion preparations could further increase the incoherence of the signal, which may improve the reconstruction. In addition, further gradient compensations could be tested in future work. Acceleration or M2,^34^ and long eddy current^82^ compensations could increase the ADC map quality. These compensations can be incorporate in the proposed approach; however, the gradient strength of our hardware (62 mT/m) was insufficient to achieve a b‐value of 500 s/mm^2^ for higher‐order moment compensations or eddy current compensations.

Finally, the proposed approach was evaluated in a small cohort of healthy subjects. Further studies on a larger cohort of healthy subjects and on patients with diffuse liver disease should be performed in future work to evaluate the potential of the proposed technique for liver disease tissue characterization and tumor staging. The validation of this novel technique in a clinical setting, in patient populations with reduce diffusivity.

## 5 CONCLUSION

The proposed MRF sequence provides a novel approach for simultaneous T_1_, T_2_, and ADC mapping in the liver in a short breath‐held scan of 16 s. The optimization of the diffusion gradient waveform provided robust ADC quantification against cardiac motion and vascular flow present during the preparation pulse. Phantom studies showed excellent correlation of T_1_, T_2_, and ADC against their respective gold standard sequences (r^2^ > 0.9 for all cases), and in vivo studies showed a good agreement between MRF‐derived and clinical reference scans. Moreover, a high in vivo intrasession repeatability was shown. Further studies are warranted to test this approach in patients with suspected diffuse liver disease to evaluate its potential for liver tissue characterization and tumor staging.

## Supporting information


**FIGURE S1.** Diffusion preparation (Dp) waveforms with different levels of optimization. Row (A) shows the standard Dp module employed in the Twice‐Refocused Spin Echo (TRSE) sequence frequently used for diffusion measurement. By definition, this Dp has null zeroth moment (MC0). (B) is a copy of TRSE with small 1‐ms gaps after each gradient block, for eddy current compensation purposes. (C) shows same Dp as in (B), with extra optimization (here denoted as MX) to compensate for concomitant gradients effect. (B) and (C) are further optimized in (D) and (E) respectively, which have been constrained to have first moment nulling (MC1) (besides zeroth moment nulling) to achieve increased robustness against blood flow and motion while the Dp is taking place. Maximum b‐value (b_max_) achieved for each Dp is shown in their top right corners, and it can be observed how b_max_ decreases when the optimization level increases. The Dp module used in the present work is shown in (E).
**FIGURE S2**. Bland–Altman plots of measured T_1_ (left), T_2_ (middle) and ADC (right) from MRF against the reference values obtained from long SE reference scans (A: IR‐SE T_1_, multiecho SE T_2_ and Diffusion Weighted SE (mSh DWI‐SE)), and shorter clinical maps that were further employed as in vivo reference scans (T_1_‐MOLLI, T_2_‐GRaSE, and DWI‐SE sSh). Quantification was performed inside circular ROIs of 15 mm diameter for every sample two of the two phantoms scanned (T1MES phantoms: blue dots, Diffusion phantom: red dots). Dashed lines represent average bias, and dotted lines denote limits of agreement (LoA), at ±1.96SD. Every Bland–Altman plot was obtained using measurements shown in Figure 6 on the main manuscript.
**FIGURE S3**. Quantitative T_1_, T_2_ and ADC maps derived from Reference (top row) and MRF scans (bottom), corresponding to (A) T1MES phantom, and (B) Diffusion phantom. Quantitative values measured within each ROI are shown in Figure 6B.
**FIGURE S4**. Individualized violin plot analysis of the test–retest repeatability performed on the 10 healthy subjects. Top, middle and bottom rows show T_1_, T_2_ and ADC quantification respectively. Each subject (V1–V10) underwent three separate MRF scans (s1, s2 and s3). T_1_, T_2_ and ADC maps were obtained for each subject and scan, and quantitative measurements inside liver were obtained within 8 circular ROIs placed in the liver. Each measurement is represented as a dot in every violin. Dashed line within the violin denotes median, and dotted lines denote 25th and 75th percentile of distribution. ROIs were spatially co‐registered for each subject among its three separate scans.
**TABLE S1**. Sequence parameters for conventional and MRF scans. * See Figure 1 for preparation pulses details of the MRF scan.** The DWI‐SE (sSh) was performed under respiratory trigger.
**TABLE S2**. Mean ± SD quantification of T_1_, T_2_ and ADC on the MRF maps averaged over the 8 ROIs of each subject from the repeatability study. Coefficient of variation (CV) as percentage, and intraclass correlation coefficient (ICC) between distributions, assuming two‐way mixed effect, absolute agreement, and multiple raters/measurements are also shown.

## Data Availability

The code to obtain the diffusion prepared gradient waveforms is readily available in https://github.com/cncastillo/DiffPrepMCMRF.
