# Supplementary material for: Simultaneous liver T1 , T2 , and ADC MR fingerprinting using optimized motion‐compensated diffusion preparations: An initial validation on volunteers
Source: Magn Reson Med. 2025 Jul 9;94(5):2173–89. doi: 10.1002/mrm.30622 (PMC12393201; doi:10.1002/mrm.30622)
Supplement: Supplementary file 1 — FIGURE S1. Diffusion preparation (Dp) waveforms with different levels of optimization. Row (A) shows the standard Dp module employed in the Twice‐Refocused Spin Echo (TRSE) sequence frequently used for diffusion measurement. By definition, this Dp has null zeroth moment (MC0). (B) is a copy of TRSE with small 1‐ms gaps after each gradient block, for eddy current compensation purposes. (C) shows same Dp as in (B), with extra optimization (here denoted as MX) to compensate for concomitant gradients effect. (B) and (C) are further optimized in (D) and (E) respectively, which have been constrained to have first moment nulling (MC1) (besides zeroth moment nulling) to achieve increased robustness against blood flow and motion while the Dp is taking place. Maximum b‐value (bmax) achieved for each Dp is shown in their top right corners, and it can be observed how bmax decreases when the optimization level increases. The Dp module used in the present work is shown in (E). FIGURE S2. Bland–Altman plots of measured T1 (left), T2 (middle) and ADC (right) from MRF against the reference values obtained from long SE reference scans (A: IR‐SE T1, multiecho SE T2 and Diffusion Weighted SE (mSh DWI‐SE)), and shorter clinical maps that were further employed as in vivo reference scans (T1‐MOLLI, T2‐GRaSE, and DWI‐SE sSh). Quantification was performed inside circular ROIs of 15 mm diameter for every sample two of the two phantoms scanned (T1MES phantoms: blue dots, Diffusion phantom: red dots). Dashed lines represent average bias, and dotted lines denote limits of agreement (LoA), at ±1.96SD. Every Bland–Altman plot was obtained using measurements shown in Figure 6 on the main manuscript. FIGURE S3. Quantitative T1, T2 and ADC maps derived from Reference (top row) and MRF scans (bottom), corresponding to (A) T1MES phantom, and (B) Diffusion phantom. Quantitative values measured within each ROI are shown in Figure 6B. FIGURE S4. Individualized violin plot analysis of the test–retest repe [file MRM-94-2173-s001.docx]

**Simultaneous Liver T_1_, T_2_ and ADC Magnetic Resonance Fingerprinting using Optimized Motion-compensated Diffusion Preparations**

C. Velasco^1,*^, C. Castillo-Passi^1,2,3,*^, N. Chaher^1^, D. C. Karampinos^4^, P. Irarrazaval^2,3,5^, A. Phinikaridou^1^, R. M. Botnar^1,2,3,5^, C. Prieto^1,3,5^

^1^School of Biomedical Engineering and Imaging Sciences, King’s College London, UK

^2^Institute for Biological and Medical Engineering, Pontificia Universidad Católica de Chile, Santiago, Chile

^3^Millenium Institute for Intelligent Healthcare Engineering, Santiago, Chile

^4^Department of Diagnostic and Interventional Radiology, School of Medicine & Klinikum rechts der Isar, Technical University of Munich, Munich, Germany

^5^School of Engineering, Electrical Engineering Department, Pontificia Universidad Católica de Chile, Santiago, Chile

*Carlos Velasco and Carlos Castillo-Passi contributed equally to this work

**Supporting Information (3 Figures and 2 Tables)**


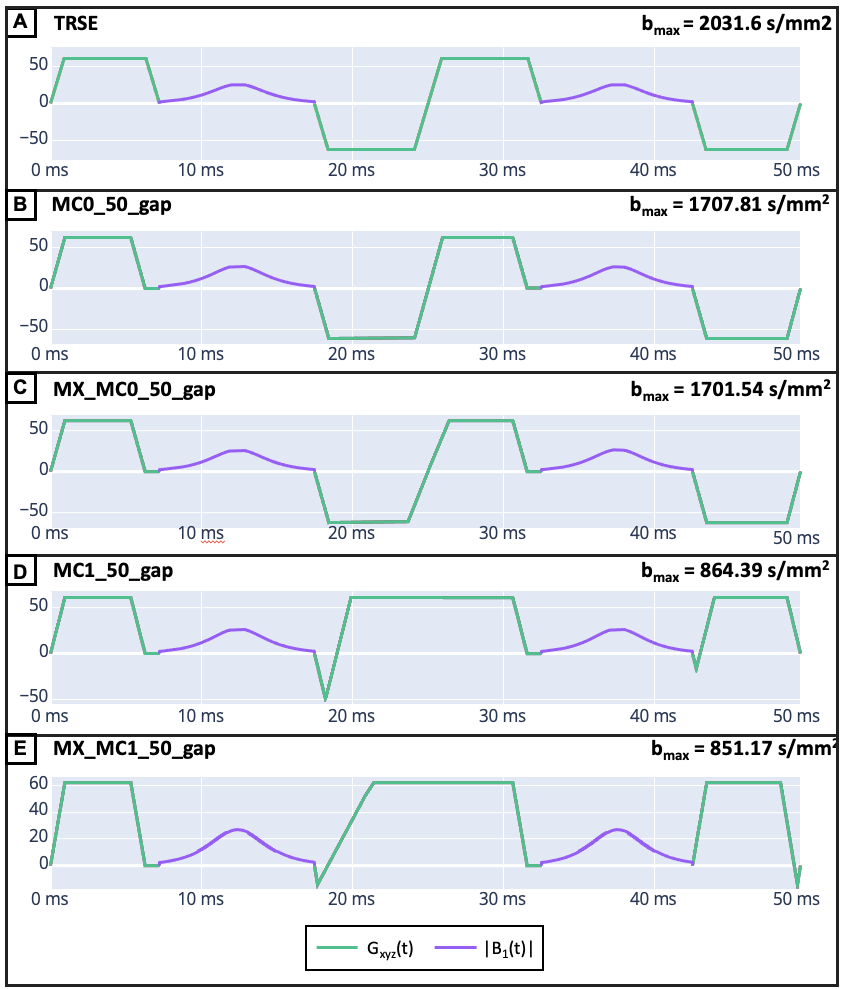


***Supporting Figure S1****. Diffusion preparation (Dp) waveforms with different levels of optimization. Row (A) shows the standard Dp module employed in the Twice-Refocused Spin Echo (TRSE) sequence frequently used for diffusion measurement. By definition, this Dp has null zero^th^ moment (MC0). (B) is a copy of TRSE with small 1-ms gaps after each gradient block, for eddy current compensation purposes. (C) shows same Dp as in (B), with extra optimization (here denoted as MX) to compensate for concomitant gradients effect. (B) and (C) are further optimized in (D) and (E) respectively, which have been constrained to have first moment nulling (MC1) (besides zeroth moment nulling) to achieve increased robustness against blood flow and motion while the Dp is taking place. Maximum b-value (b_max_) achieved for each Dp is shown in their top right corners, and it can be observed how b_max_ decreases when the optimization level increases. The Dp module used in the present work is shown in (E).*


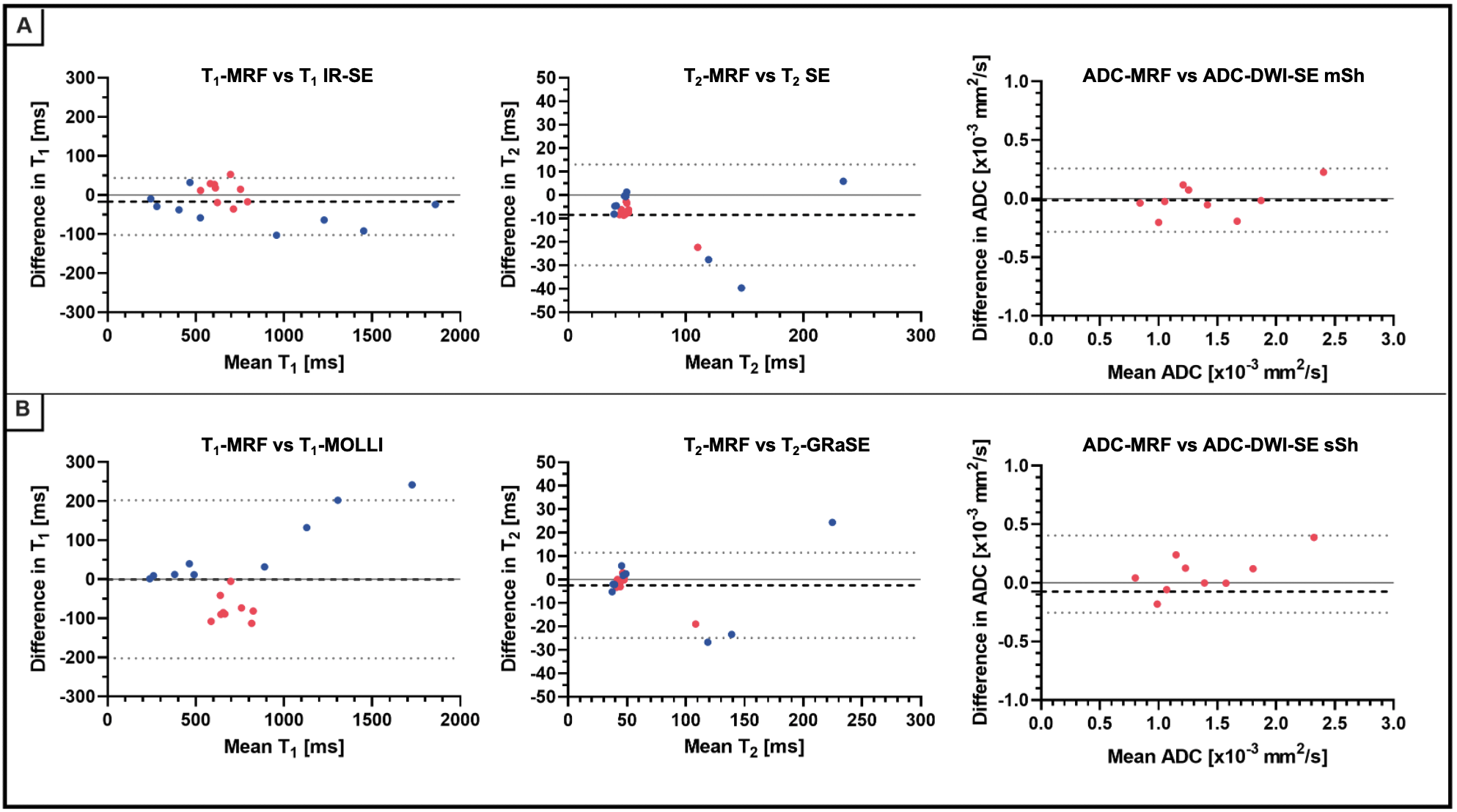


***Supporting Figure S2****. Bland-Altman plots of measured T_1_ (left), T_2_ (middle) and ADC (right) from MRF against the reference values obtained from long SE reference scans (A: IR-SE T_1_, multiecho SE T_2_ and Diffusion Weighted SE (multishot DWI-SE)), and shorter clinical maps that were further employed as in vivo reference scans (T_1_-MOLLI, T_2_-GRaSE, and DWI-SE sSh). Quantification was performed inside circular ROIs of 15mm diameter for every sample two of the two phantoms scanned (T1MES phantoms: blue dots, Diffusion phantom: red dots). Dashed lines represent average bias, and dotted lines denote limits of agreement (LoA), at ± 1.96SD. Every Bland-Altman plot was obtained using measurements shown in Figure 6 on the main manuscript.*


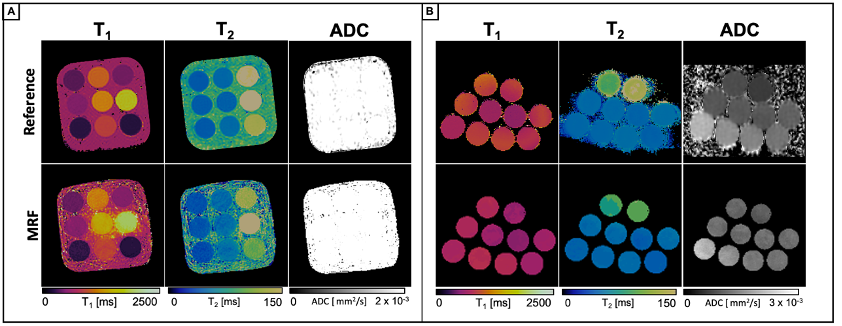


***Supporting Figure S3****. Quantitative T_1_, T_2_ and ADC maps derived from Reference (top row) and MRF scans (bottom), corresponding to (A) T1MES phantom, and (B) Diffusion phantom. Quantitative values measured within each ROI are shown in Figure 6B.*

***Supporting Figure S4****. Individualised violin plot analysis of the test-retest repeatability performed on the 10 healthy subjects. Top, middle and bottom rows show T_1_, T_2_ and ADC quantification respectively. Each subject (V1-V10) underwent three separate MRF scans (s1, s2 and s3). T_1_, T_2_ and ADC maps were obtained for each subject and scan, and quantitative measurements inside liver were obtained within 8 circular ROIs placed in the liver. Each measurement is represented as a dot in every violin. Dashed line within the violin denotes median, and dotted lines denote 25^th^ and 75^th^ percentile of distribution. ROIs were spatially co-registered for each subject amongst its three separate scans.*

|  | **T_1_-IRSE** | **T_1_-MOLLI** | **T_2_-MESE** | **T_2_-GraSE** | **DWI -SE (mSh)** | **DWI -SE (sSh)** | **MRF** |
| --- | --- | --- | --- | --- | --- | --- | --- |
| FOV (mm × mm) | 256 × 256 | 320 × 280 | 256 × 256 | 320 × 320 | 224 × 224 | 320 × 320 | 320 × 320 |
| Acq. Voxel (mm × mm) | 1 × 1 | 2 × 2 | 1 × 1 | 2 × 2 | 2 × 2 | 3 × 3 | 1 × 1 |
| Slice Thickness (mm) | 6 | 6 | 6 | 6 | 8 | 4 | 6 |
| TR (ms) | 5000 | 2.4 | 5000 | 984 | 800 | 2686 | 7.0 |
| TE (ms) | 20 | 1.13 | 11, 43, 65, 75, 97 | 20 + 9.8n | 50 | 55 | 3.3 |
| FA (ˇ) | 90 | 20 | 90 | 90 | 90 | 90 | 10-20 |
| TI (ms) | 120, 200, 600, 800, 1000, 1500, 1700, 2000 | 350 | N/A | N/A | N/A | N/A | * |
| b-val (s/mm^2^) | N/A | N/A | N/A | N/A | 0(1), 100(2), 200(2), 300(2), 400(2), 500(2), 600(3), 700(3) | 0(1), 50(1), 300(2), 600(3), 800(4) | * |
| Scan time | 42min | 13 s | 15 min | 14 s | 4 min 36s | 1 min40s** | 16 6s |

**Supporting Table S1**. Sequence parameters for conventional and MRF scans. * See Figure 1 for preparation pulses details of the MRF scan.** The DWI-SE (sSh) was performed under respiratory trigger.

|  | **Scan #1** | **Scan #2** | **Scan #3** | **CV (%)** | **ICC** |
| --- | --- | --- | --- | --- | --- |
| MRF-T_1_ (ms) | 841.3 ± 71.0 | 827.8 ± 81.4 | 826.4 ± 84.4 | 2.61 | 0.970 |
| MRF-T_2_ (ms) | 36.80 ± 3.22 | 36.52 ± 3.55 | 37.49 ± 3.07 | 3.89 | 0.915 |
| MRF-ADC (mm^2^/s) | 0.97 ± 0.13 | 0.955 ± 0.134 | 0.975 ± 0.113 | 2.67 | 0.952 |

**Supporting Table S2**. Mean ± SD quantification of T_1_, T_2_ and ADC on the MRF maps averaged over the 8 ROIs of each subject from the repeatability study. Coefficient of variation (CV) as percentage, and intraclass correlation coefficient (ICC) between distributions, assuming two-way mixed effect, absolute agreement, and multiple raters/measurements are also shown.
